# Grape Seed Extract Mitigates Acute Stress-Induced Neuroinflammation and Oxidative Damage in Female Mice: Evidence for Gut–Brain Axis Modulation

**DOI:** 10.1007/s11064-026-04939-9

**Published:** 2026-10-27

**Authors:** Rafaela Xavier, Marina Rigotti, Renata L. De Oliveira, Laura F. Finger, Lauren N. Pujol, Fernanda S. S. Sousa, Suzana L. Lanius, Tiago V. Collares, Fabiana K. Seixas, Catia S. Branco, Lucielli Savegnago

**Affiliations:** 1 Technology Development Center, Biotechnology Unit, Neurobiotechnology Research Group, Federal University of Pelotas, Pelotas, RS Brazil; 2 Oxidative Stress & Antioxidants Laboratory, Institute of Biotechnology, University of Caxias do Sul, Caxias do Sul, RS Brazil; 3 Technology Development Center, Molecular and Cellular Oncology Research Group, Cancer Biotechnology Laboratory, Federal University of Pelotas, Pelotas, RS Brazil

**Keywords:** Depression, Grape seed extract, Corticosterone, Antioxidant, Neuroinflammation

## Abstract

Short-term stress is known to trigger oxidative and neuroinflammatory responses that contribute to the onset and progression of major depressive disorder (MDD). Phenolic compounds, such as grape seed extract (GSE), have gained attention for their ability to modulate these biological pathways. This study aimed to evaluate whether GSE (20 or 40 mg/kg, oral route) exerts antidepressant-like effects and mitigates stress-induced biochemical alterations in female mice exposed to the acute restraint stress (ARS) model. Female mice were pretreated with GSE and subsequently subjected to ARS. Behavioral outcomes were assessed using the tail suspension test (TST) and open field test (OFT). Plasma corticosterone levels, reactive species (RS) production in the prefrontal cortex, hippocampi, and small intestine, and mRNA expression of inflammatory mediators (NF-κB, IL-1β, IFN-γ) in brain and gut tissues were quantified through standard biochemical and molecular analyses. GSE pretreatment significantly prevented the ARS-induced increase in immobility time in the TST, without affecting locomotor activity in the OFT. ARS exposure elevated corticosterone concentrations and RS generation across central and peripheral tissues; both effects were attenuated by GSE. Additionally, GSE downregulated stress-induced expression of pro-inflammatory cytokines in neural and intestinal samples, indicating suppression of key inflammatory pathways. These findings demonstrate that GSE exerts antidepressant-like, antioxidant, and anti-inflammatory effects through interconnected redox and cytokine signaling pathways, as summarized in the Graphical Abstract. Importantly, this study provides novel evidence that GSE acts along the gut–brain axis, mitigating corticosterone-driven oxidative and inflammatory responses. These results highlight GSE as a promising natural compound for the prevention and management of stress-related neuropsychiatric disorders.

**Graphical Abstract:**

**Figure:**
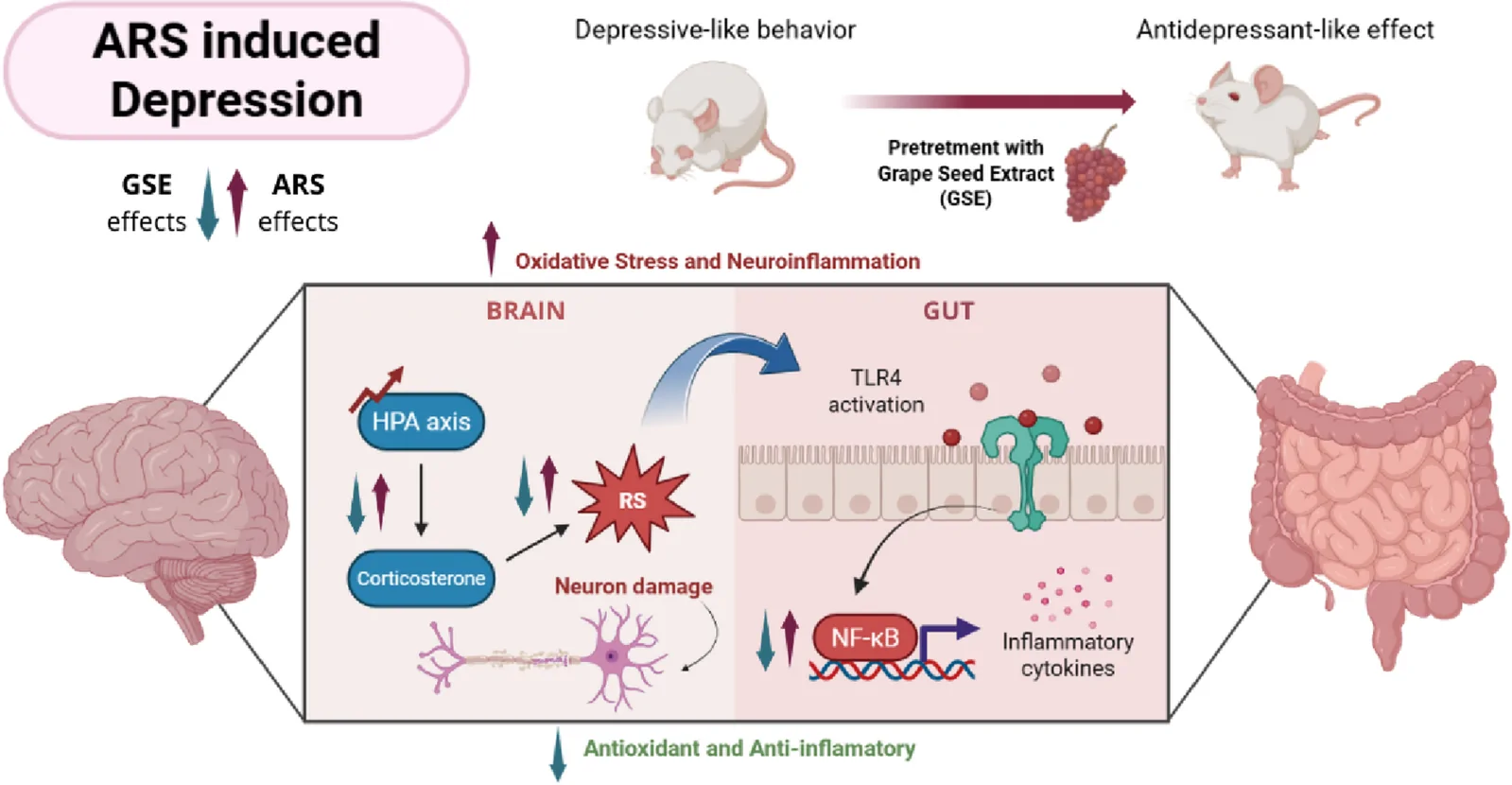
Graphical abstract summarizing the antidepressant-like, antioxidant, and anti-inflammatory effects of grape seed extract (GSE) in mice subjected to acute restraint stress (ARS)

## Introduction

Grapes (*Vitis vinifera*) are among the most widely consumed fruits globally, and they are a rich source of bioactive compounds, particularly polyphenols with potent antioxidant activity. Grape seed extract (GSE) contains over 90% polyphenols, including both flavonoid and non-flavonoid constituents [1]. It is primarily composed of flavan-3-ol derivatives, such as catechin and epicatechin, in the form of monomers, dimers, trimers, and oligomers [2].

As a natural co-product derived from grape, GSE has gained attention as a sustainable source of bioactive compounds with multiple therapeutic properties [3, 4] Given the strong interplay between chronic inflammation and pathological processes such as cancer, neurodegeneration, and mood disorders, the antioxidant and anti-inflammatory effects of GSE have broad translational relevance. Its unique phytochemical composition underlies diverse pharmacological actions, particularly in the prevention and management of neurodegenerative and mood disorders [5–8] These properties are particularly important since oxidative and neuroinflammatory processes are key mechanisms driving major depressive disorder (MDD), often triggered by acute stress [9, 10].

MDD is a multifactorial psychiatric condition characterized by persistent low mood, anhedonia, and disturbances in sleep, appetite, energy, and cognition [11]. Its complex etiology involves the interplay between neuroinflammation, oxidative stress, and neuroendocrine dysregulation, which together impair neurotransmission and synaptic plasticity [12]. According to the World Health Organization (WHO, 2025), MDD affects women almost twice as often as men, a disparity that emerges after puberty and persists throughout the reproductive years, underscoring the importance of investigating sex-specific mechanisms and therapeutic responses [13]. This increased susceptibility is thought to result from a complex interaction among sex hormones, stress responsiveness, immune regulation, and neurobiological mechanisms [14]. Experimental studies have demonstrated that fluctuations in ovarian hormones influence HPA axis activity, neuroplasticity, and inflammatory signaling, rendering females more vulnerable to stress-induced mood disorders [14, 15]. Despite these well-established sex differences, females remain underrepresented in preclinical studies, highlighting the need for experimental investigations specifically addressing the biological mechanisms underlying depression in females [13, 16, 17].

Exposure to stress activates the hypothalamic–pituitary–adrenal (HPA) axis, leading to the excessive release of glucocorticoids, mainly cortisol, from the adrenal cortex. Sustained glucocorticoid elevation promotes receptor resistance and disrupts the negative feedback regulation of the HPA axis [12]. This dysregulation has profound metabolic and neurobiological consequences, including enhanced mitochondrial activity and overproduction of reactive oxygen species (ROS). Elevated ROS levels activate microglia and astrocytes, triggering the release of proinflammatory cytokines and mediators that perpetuate neuroinflammation and oxidative imbalance within the central nervous system (CNS) [18].

Previous studies have shown that GSE exerts neuroprotective effects by modulating pathways associated with neuroinflammation and oxidative stress. Sun [1] demonstrated that GSE administration reduced hippocampal oxidative stress and neuroinflammation in prenatally stressed female rats, accompanied by attenuation of depressive-like behaviors. Similarly, Jiang [19] reported that GSE pretreatment mitigated depressive behavior and suppressed the expression of inflammatory markers such as interleukin-1β (IL-1β) and nuclear factor kappa B (NF-κB) in mice challenged with lipopolysaccharide. In vitro, GSE reestablished redox balance and suppressed NLRP3-mediated inflammation induced by the tryptophan catabolite quinolinic acid in a glial microenvironment. Collectively, these findings indicate that the antidepressant-like effects of GSE are closely linked to its ability to modulate oxidative and inflammatory signaling pathways.

Given this background, the present study aims to evaluate the potential antidepressant-like effects of GSE pretreatment in female Swiss mice subjected to the acute restraint stress (ARS) model. By assessing behavioral, oxidative, and inflammatory parameters, this study seeks to elucidate the mechanisms through which GSE may modulate HPA axis dysfunction and exert neuroprotective and anti-inflammatory actions in stress-induced depressive-like states.

## Materials and Methods

### Animals

Experiments were conducted using female Swiss mice, aged between 4 and 6 weeks (20–25 g), obtained from the Central Animal Facility of the Federal University of Pelotas (UFPel). Animals were housed in groups of seven per cage (*n* = 7 animals per experimental group) under standard environmental conditions (22 ± 1 °C; 12 h light/dark cycle) with free access to food and water. The number of animals was defined according to the experimental design and in accordance with the ethical recommendations for animal experimentation, considering the principles of reduction and refinement. All experimental procedures were performed between 08:00 a.m. and 06:00 p.m., in accordance with the guidelines of the UFPel Animal Ethics Committee (CEUA/UFPel, protocol no. 131/2023).

### Grape Seed Extract

The GSE was purchased from Xi’an Herbspirit Technology Co., Ltd. (Batch No. XC20230310, Shaanxi, P.R. China). The phenolic characterization and composition of the extract were previously described by Rigotti et al. [7]. For animal use, the extract was diluted in distilled water to final concentrations of 20 or 40 mg/kg and filtered using a sterile syringe filter (polyethersulfone, 0.22 μm pore size). The extract doses and pretreatment period were established based on the previous study by Jiang et al. [19]. Animals received daily intragastric (i.g.) administration of GSE or vehicle (distilled water) for seven consecutive days at doses of 20 or 40 mg/kg.

### Acute Restraint Stress (ARS)

The animals were submitted to physical restraint as previously reported by Domingues et al. [20]. Mice were subjected to immobilization for 4 h using an individual rodent restraint device made of fenestrated Plexiglas, restraining physical movement. During the period of exposure to stress, the animals were deprived of water and food. After the 4-hours period, the animals were returned to their respective home cages.

### Experimental Design

**Fig. 1:**
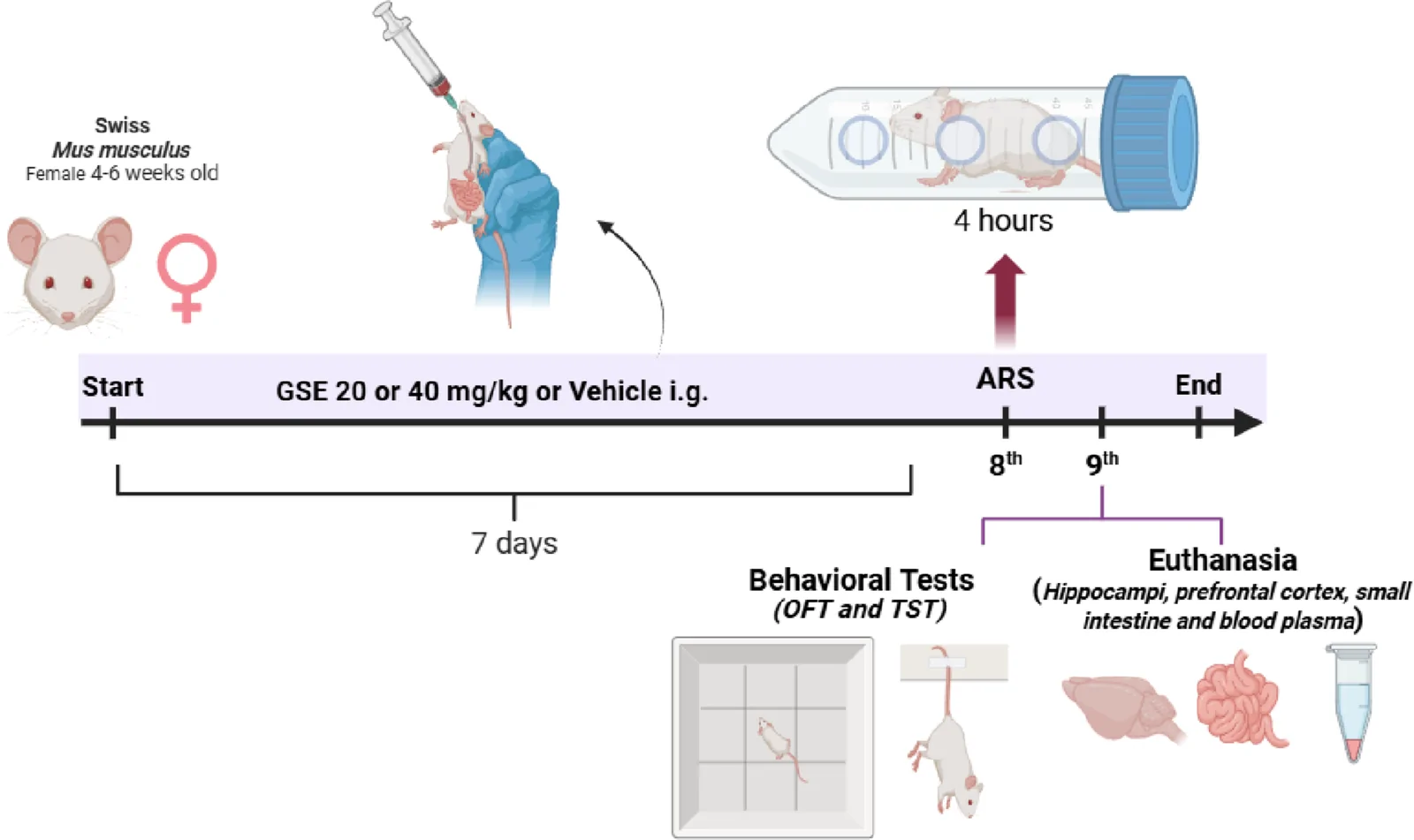
Experimental design. GSE: Grape seed extract; ARS: Acute restraint stress; OFT: Open field test; TST: Tail suspension test

The experimental design is illustrated in Fig. 1. This study aimed to investigate the potential of GSE to prevent ARS-induced depressive-like behavior. Animals were randomly assigned to six experimental groups (*n* = 7 per group):

- G1: Non-stressed Control (No stress + vehicle).
- G2: GSE 20 mg/kg per se (No stress + GSE 20).
- G3: GSE 40 mg/kg per se (No stress + GSE 40).
- G4: ARS-induced (ARS + vehicle).
- G5: GSE 20 mg/kg (ARS + GSE 20).
- G6: GSE 40 mg/kg (ARS + GSE 40).

Animals received daily intragastric (i.g.) administration of GSE or vehicle (distilled water) for seven consecutive days at doses of 20 or 40 mg/kg. This pretreatment regimen was designed to evaluate the preventive effects of GSE against ARS-induced behavioral and biochemical alterations. On the eighth day, mice were subjected to 4 h of ARS and were subsequently returned to their home cages. On the ninth day, approximately 24 h after the end of ARS exposure, behavioral assessments were performed using the Open Field Test (OFT) and the Tail Suspension Test (TST). Immediately after completion of the behavioral tests, animals were anesthetized with isoflurane (inhalation) for cardiac puncture and blood collection to determine plasma corticosterone levels. The prefrontal cortex, hippocampi, and small intestinal tissues were also collected for subsequent biochemical and molecular analyses.

### Behavioral Tests

#### Open Field Test (OFT)

The OFT was conducted prior to other behavioral assessments, following the procedure described by Walsh and Cummins [21]. This test measures behavioral changes in rodents exposed to novel environments and helps to confirm that depression-like behavior is not due to general motor activity alterations . Each mouse was individually placed in the center of an open field box (30 × 30 × 15 cm) divided into nine equal quadrants and observed for 5 min. The locomotor activity was quantified by the number of quadrants crossed, while the exploratory behavior, by the number of rearing events.

#### Tail Suspension Test (TST)

The total immobility time during the TST was measured as described by Steru et al. [22]. Mice were individually suspended 50 cm above the floor using adhesive tape placed approximately 1 cm from the tip of the tail. The immobility time, defined as the absence of escape-oriented movements, was recorded during the last 4 min of a 6-minutes session. The test was conducted manually by a blind observer to the experimental conditions.

### Biochemical Analyses

#### Tissue Preparation

After behavioral tests, animals were anesthetized with isoflurane inhalation, and blood was collected by cardiac puncture into heparinized tubes. Samples were centrifuged at 3,500 rpm for 10 min, and the plasma was used for corticosterone quantification. Following blood collection, animals were euthanized by decapitation, and the prefrontal cortex, hippocampi and small intestine were rapidly dissected and kept on ice until homogenization in 50 mM Tris-HCl buffer (pH 7.4; 1:10 w/v). The homogenates were centrifuged at 2,500 ×g for 10 min at 4 °C, and the supernatant (S1 fraction) was used for reactive species (RS) assay. For gene expression analysis, a separate group of animals was used; their prefrontal cortex, hippocampi, and small intestinal samples were stored in Trizol reagent at − 80 °C for subsequent qRT-PCR analysis.

#### Plasma Corticosterone Levels

Corticosterone levels were determined according to Zenker and Bernstein [23]. Briefly, heparinized blood was centrifuged at 3,500 rpm for 10 min to obtain plasma. To 200 µL of plasma, 800 µL of distilled water and 2 mL of chloroform were added, mixed for 15 s, and centrifuged at 2,500 rpm for 5 min. The aqueous phase was discarded, and the chloroform phase was retained. Then, 1 mL of 0.1 M NaOH was added, mixed for 15 s, and centrifuged again. One milliliter of the chloroform phase was transferred to a new tube, mixed with 3 mL of fluorescence reagent, and centrifuged once more. The chloroform phase was then incubated for 2 h before spectrofluorometric reading (excitation = 247 nm; emission = 540 nm; slit = 5 nm). Plasma corticosterone levels were expressed as ng/mL.

#### Reactive Species (RS)

RS quantification in prefrontal cortex, hippocampi, and small intestine of pretreated mice followed the method described by Loetchutinat et al. [24]. Briefly, 10 µL of 1 mM DCFH-DA (2’,7’-dichlorodihydrofluorescein diacetate) were incubated with 10 µL of tissue homogenate and 2,990 µL of 10 mM Tris-HCl buffer (pH 7.4). The oxidation of DCFH-DA to fluorescent DCF was used as an indicator of intracellular RS. Fluorescence intensity was measured at 480 nm excitation and 520 nm emission, and results were expressed as fluorescence units per mg of protein.

#### Protein Quantification

Protein content was determined by the Bradford [25] method using bovine serum albumin (1 mg/mL) as the standard. After sample homogenization, 5 µL of homogenate were incubated with 45 µL of distilled water and 2.5 mL of Coomassie Brilliant Blue reagent for 10 min, and absorbance was read at 595 nm using a spectrophotometer.

### RNA Extraction and Gene Expression Analysis by qRT-PCR

Total mRNA was extracted from the cortex, hippocampi, and small intestine using TRIzol followed by mRNA quantification. The cDNA synthesis was performed using a High-Capacity cDNA Reverse Transcription kit according to the manufacturer’s protocol. The amplification was made with the GoTaq^®^ qPCR Master Mix using the Agilent AriaMX real-time PCR. Gene expressions were normalized using GAPDH primer as a reference gene. The 2ΔΔCT (Delta–Delta Comparative Threshold) method was used to normalize the fold change in gene expression. The genes analyzed are shown in Table 1.

Based on the comparable behavioral and biochemical efficacy observed for the 20 and 40 mg/kg GSE doses, qRT-PCR analyses were performed only in the 20 mg/kg group, as this represented the lowest effective dose. This approach is commonly adopted in preclinical pharmacological studies to investigate the mechanisms underlying the therapeutic effects while minimizing unnecessary experimental complexity.

**Table 1:** Primer sequences used

Gene	Sequence 5’-3’	Amplicon size (bp)	Tₐ (°C)
GAPDH	F: GGGTGAGGCCGGTGCTGAG R: TGGGGGTAGGAACACGGAAGG	150	60
IL-1β	F: GCTGAAAGCTCTCCACCTCAATG R: TGTCGTTGCTTGGTTCTCCTTG	140	60
IFN-γ	F: GCGTCATTGAATCACACCTG R: TGAGCTCATTGAATGCTTGG	120	60
NF-κB	F: GCTTTCGCAGGAGCATTAAC R: CCGAAGCAGGAGCTATCAAC	130	60

### Statistical Analysis

Data were analyzed using two-way ANOVA followed by Tukey’s post hoc test, performed with GraphPad Prism 8.0 software (San Diego, CA, USA). Results are expressed as mean ± standard error of the mean (S.E.M), and values < 0.05 (*p* < 0.05) were considered statistically significant.

## Results

### GSE Attenuated ARS‑Induced Depressive‑Like Behavior Without Altering Locomotor and Exploratory Activity

**Fig. 2:**
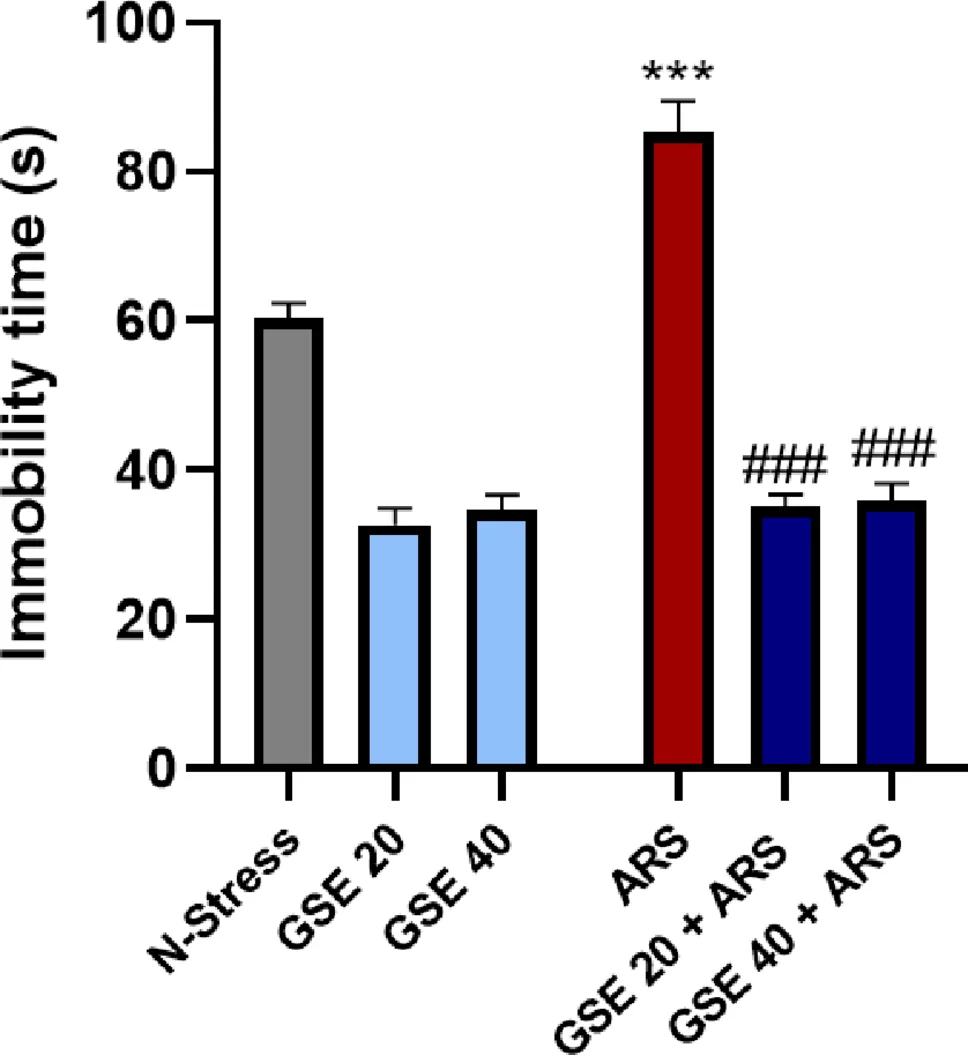
Effect of pretreatment with GSE (20 and 40 mg/kg) in female mice subjected to ARS on the tail suspension test (TST). Data are expressed as mean ± standard error of the mean (SEM, *n* = 7). ***p < 0.001 when compared with the control group, and ^###^*p* < 0.001 when compared with the ARS group. Statistical analyses were performed using two-way ANOVA followed by Tukey’s post hoc test

The effect of GSE pretreatment on immobility time in the TST is shown in Fig. 2. The two-way ANOVA followed by Tukey’s post hoc test revealed that mice subjected to ARS exhibited a significant increase in immobility time compared to the non-stressed control group, indicating depressive-like behavior. Pretreatment with GSE at both doses (20 and 40 mg/kg) significantly attenuated the ARS-induced increase in immobility time, demonstrating an antidepressant-like effect. Notably, GSE per se did not increase immobility time in the TST and exhibited even lower values than those observed in the non-stressed control group (ANOVA: F_(2,36)_ = 14.22, *p* < 0.001).

**Fig. 3:**
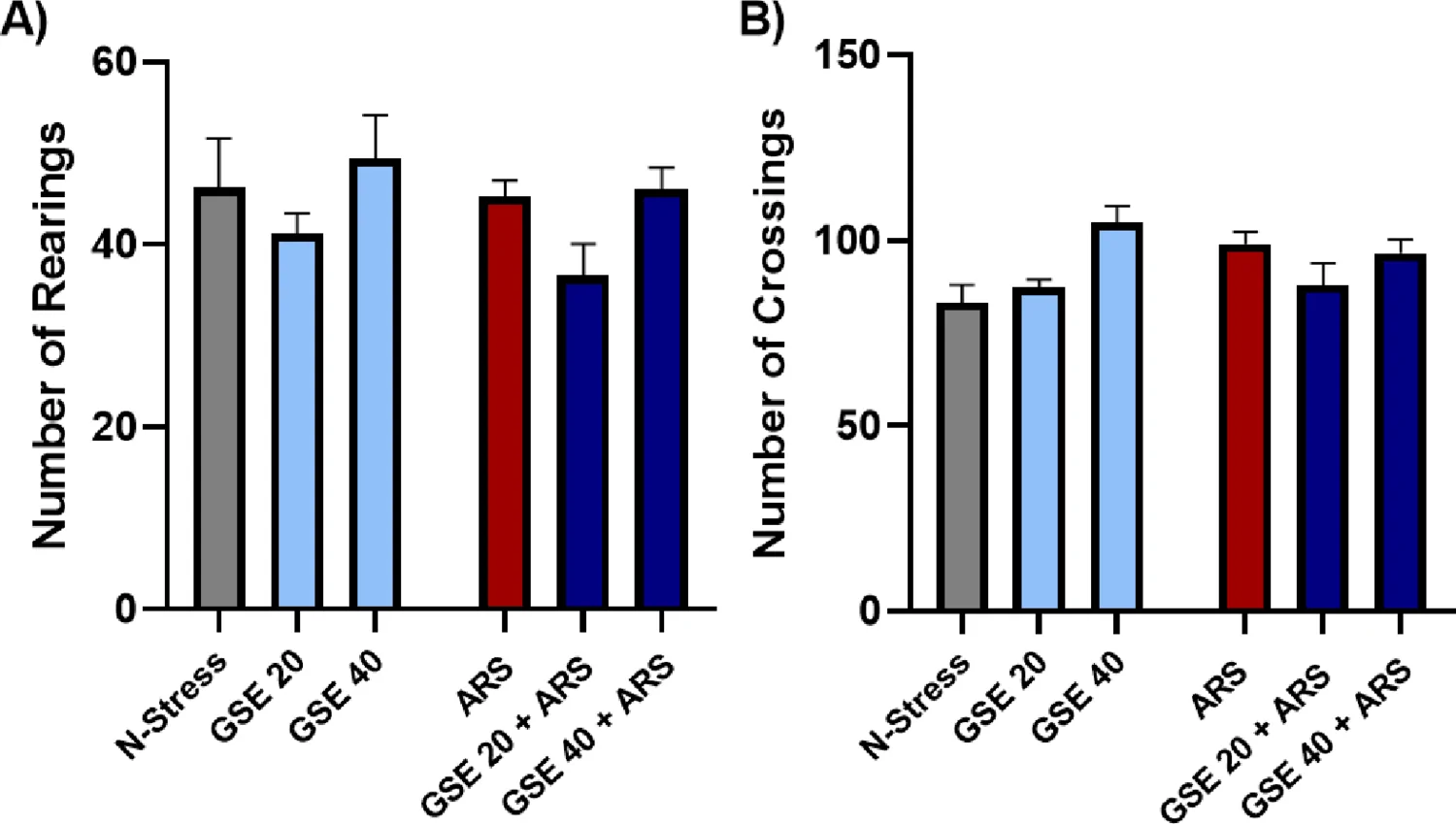
Effect of pretreatment with GSE (20 and 40 mg/kg) in female mice subjected to ARS on the OFT parameters of rearings (**A**) and crossings (**B**). Data are expressed as mean ± standard error of the mean (SEM, *n* = 7). Statistical analyses were performed using two-way ANOVA followed by Tukey’s post hoc test

The data analysis of OFT showed no changes in the number of rearings (Fig. 3A) (ANOVA: F_(2,36)_ = 0.1297, *p* = 0.88) and crossings (Fig. 3B) (ANOVA: F_(2,36)_ = 4.089, *p* = 0.03) after the different pretreatments in mice.

### GSE Attenuated Changes in Corticosterone Levels Caused by ARS-Induction

**Fig. 4:**
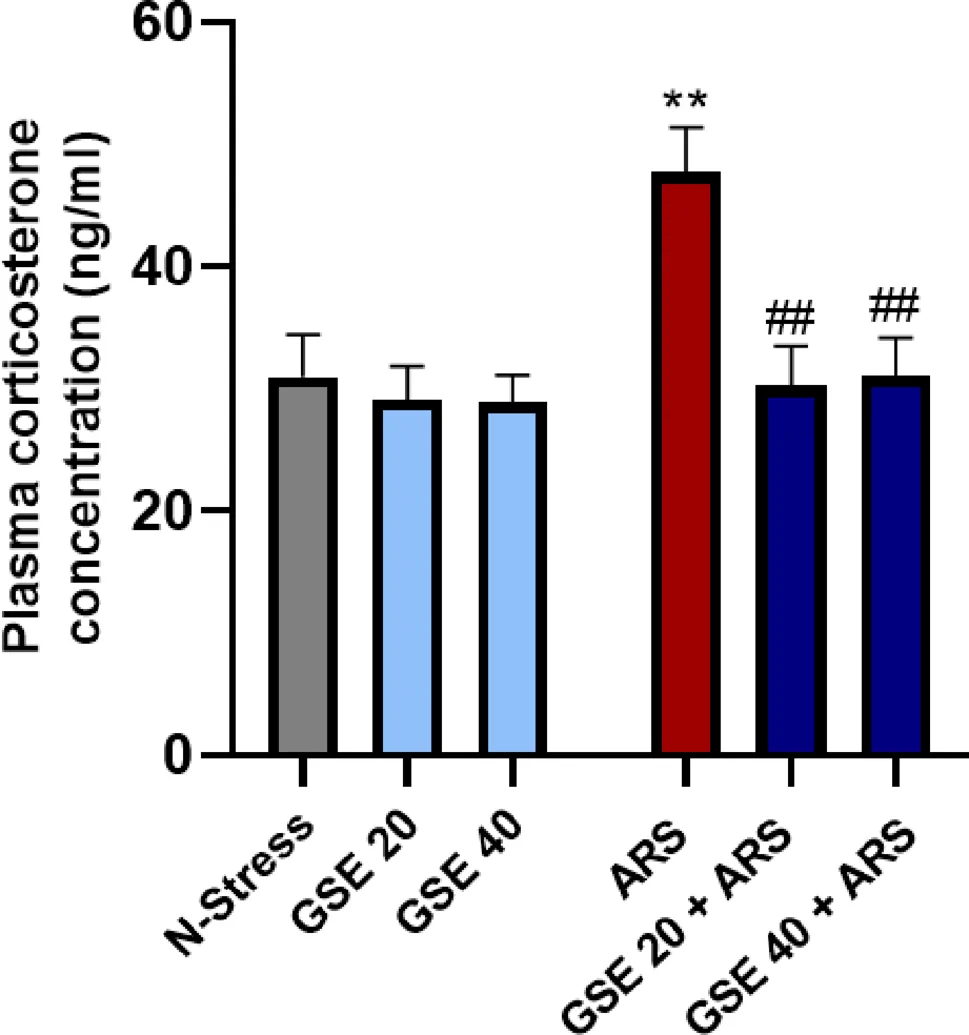
Effect of pretreatment with GSE (20 and 40 mg/kg) in female mice subjected to ARS on the corticosterone levels on blood plasma. Data are expressed as mean ± standard error of the mean (SEM, *n* = 7). **p < 0.01 when compared with the control group, and ^##^*p* < 0.01 when compared with the ARS group. Statistical analyses were performed using two-way ANOVA followed by Tukey’s post hoc test

The two-way ANOVA followed by Tukey’s post-hoc test revealed that ARS increased circulating corticosterone levels of mice, when compared to the non-stressed control group. Pretreatment with GSE at both doses (20 and 40 mg/kg) normalized these levels (Fig. 4). GSE per se did not change the circulating corticosterone levels in mice. (ANOVA: F_(2,36)_ = 3.904, *p* = 0.03).

### GSE Reduced Oxidative Damage in the Prefrontal Cortex, Hippocampi, and Small Intestine of Stressed Mice

**Fig. 5:**
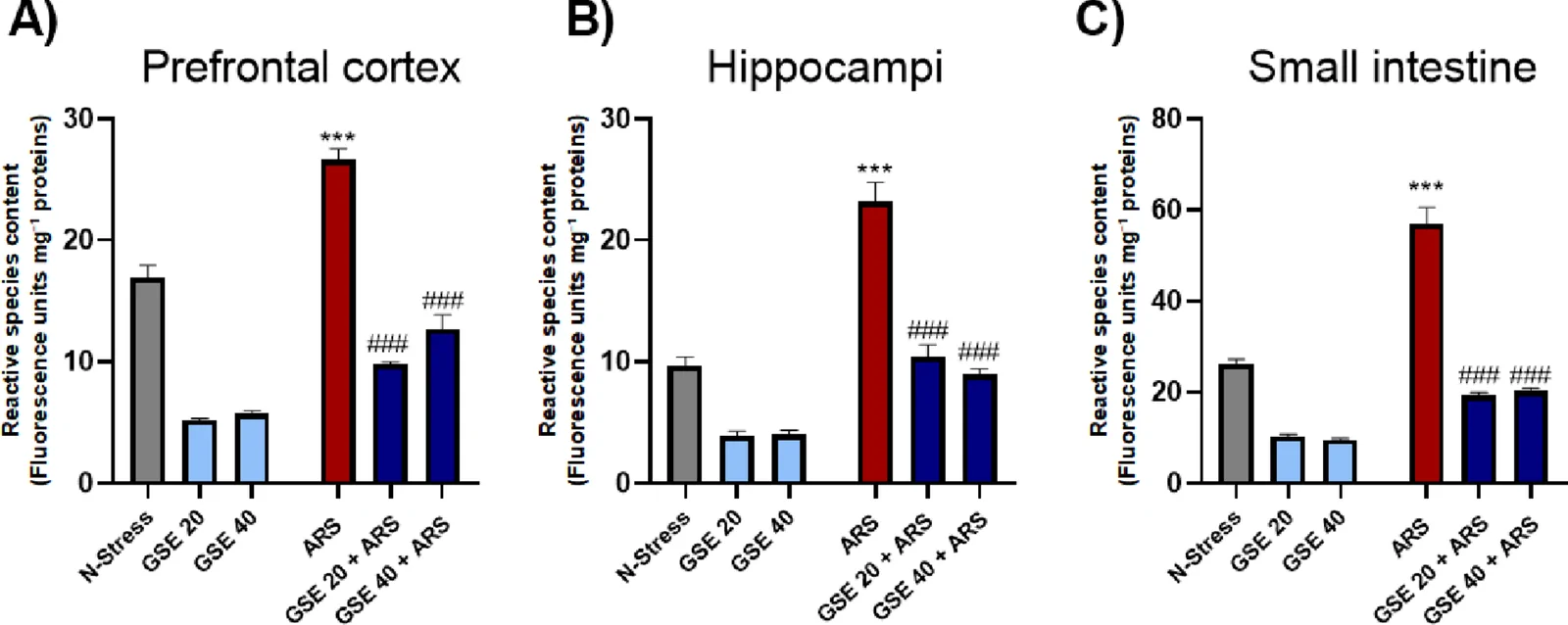
Effect of pretreatment with GSE (20 and 40 mg/kg) in female mice subjected to ARS on the reactive species test (RS) on prefrontal cortex (**A**), hippocampi (**B**) and small intestine (**C**). Data are expressed as mean ± standard error of the mean (SEM, *n* = 7). ***p < 0.001 when compared with the control group; ^###^*p* < 0.001 when compared with the ARS group. Statistical analyses were performed using two-way ANOVA followed by Tukey’s post hoc test

Figure 5 illustrates RS levels in the prefrontal cortex, hippocampi, and small intestine of mice. ARS increased RS levels in prefrontal cortex (Fig. 5A), hippocampi (Fig. 5B) and in the small intestine (Fig. 5C) of mice, when compared to the non-stressed control group. Pretreatment with GSE at both doses (20 and 40 mg/kg) significantly reduced the production of RS caused by ARS in the cerebral structures and small intestine (Fig. 5). GSE per se did not increase RS levels in the prefrontal cortex, hippocampi, or small intestine, displaying values lower than those observed in the non-stressed control group. (ANOVA: F_(2,36)_ = 5.999, *p* = 0.006 for prefrontal cortex; ANOVA: F_(2,36)_ = 13.77, *p* < 0.001 for hippocampi; ANOVA: F_(2,36)_ = 27.85, *p* < 0.001 for small intestine).

### GSE Reduced Levels of IL-1β, IFN-γ and NF-κB in the Prefrontal Cortices, Hippocampi and Small Intestine of Stressed Mice

**Fig. 6:**
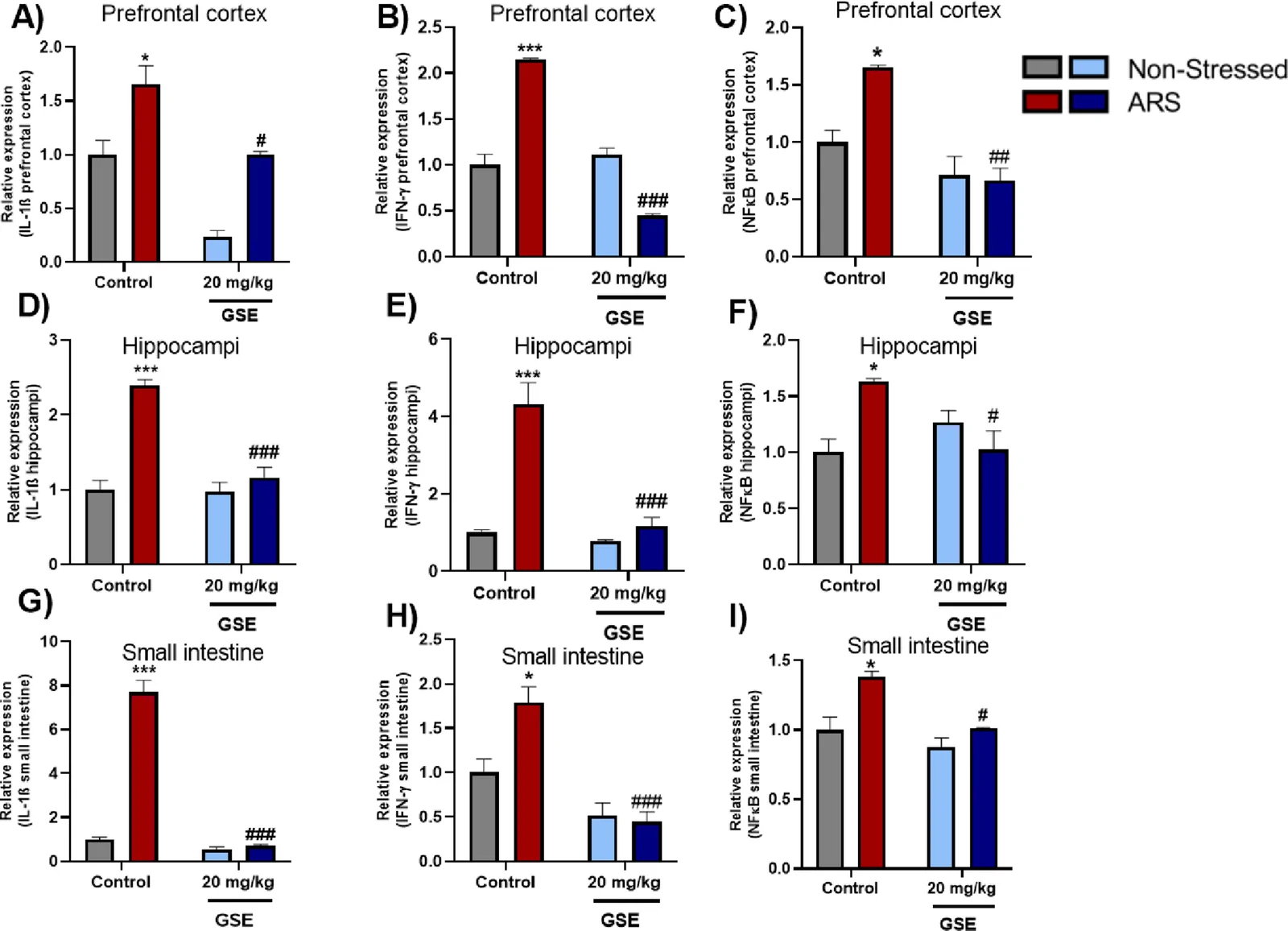
Effect of pretreatment with GSE (20 mg/kg) on the relative expression of pro-inflammatory genes in different regions of female mice subjected to ARS on prefrontal cortex (**A**, **B**, and **C**) hippocampi (**D**, **E** and **F**) and small intestine (**G**, **H** and **I**) Data are expressed as mean ± standard error of the mean (SEM, *n* = 3). *p < 0.05, ***p < 0.001 when compared with the control group; #*p* < 0.05, ##*p* < 0.01 and ###*p* < 0.001 when compared with the ARS group. Statistical analyses were performed using two-way ANOVA followed by Tukey’s post hoc test

Figure 6 presents the relative mRNA expression of inflammatory markers in the prefrontal cortex, hippocampi, and small intestine, as assessed by qRT-PCR. ARS increased IL-1β expression in the prefrontal cortex (Fig. 6A), hippocampi (Fig. 6D) and small intestine (Fig. 6G), when compared to the non-stressed control group. Post hoc comparisons showed that pretreatment with GSE at 20 mg/kg significantly reduced IL-1β mRNA expression in stressed mice in the prefrontal cortex, hippocampi, and small intestine of stressed mice. However, the two-way ANOVA did not reveal a significant treatment × ARS interaction for IL-1β expression in the prefrontal cortex (ANOVA: F_(1,8)_ = 0.2241, *p* = 0.65), indicating that the interaction between these factors was not statistically significant in this region. In contrast, significant effects were observed in the hippocampi (ANOVA: F_(1,8)_ = 26.3, *p* < 0.001) and small intestine (ANOVA: F_(1,8)_ = 135.00, *p* < 0.001). No significant changes in IL-1β mRNA expression were observed following GSE pretreatment in the absence of ARS (per se condition) in the brain structures or small intestine.

Interferon-gamma (IFN-γ) expressions in the prefrontal cortex, hippocampi and small intestine of mice are shown in Fig. 6B, E and H, respectively. ARS increased *IFN-γ* expression in the prefrontal cortex (Fig. 6B), hippocampi (Fig. 6E) and small intestine (Fig. 6H), when compared to the non-stressed control group. Pretreatment with GSE at dose of 20 mg/kg normalized the mRNA expression levels of *IFN-γ* in the prefrontal cortex, hippocampi and small intestine of stressed mice. No changes in the *IFN-γ* expression were observed after per se pretreatment with GSE in the evaluated structures (ANOVA: F_(1,8)_ = 169.5, *p* < 0.001 for prefrontal cortex; ANOVA: F_(1,8)_ = 23.65, *p* = 0.001 for hippocampi; ANOVA: F_(1,8)_ = 8.518, *p* = 0.02 for small intestine).

ARS increased the mRNA expression levels of NF-κB in the prefrontal cortex (Fig. 6C), hippocampi (Fig. 6F) and small intestine (Fig. 6I), when compared to the non-stressed control. Pretreatment with GSE at dose of 20 mg/kg normalized this increase in the structures evaluated. GSE per se did not change the mRNA expression levels of NF-κB in the prefrontal cortex, hippocampi of mice (ANOVA: F_(1,8)_ = 9.885, *p* = 0.01 for prefrontal cortex; ANOVA: F_(1,8)_ = 14.89, *p* = 0.005 for hippocampi). Although post hoc comparisons indicated differences between specific experimental groups, the treatment × ARS interaction for intestinal NF-κB expression did not reach statistical significance (ANOVA: F_(1,8)_ = 3.763, *p* = 0.0884).

## Discussion

The present study demonstrates that the pretreatment with GSE effectively preventedthe behavioral alterations induced by restraint stress in the TST, without impairing locomotion in the OFT. These findings indicate that the antidepressant-like effects of GSE are likely related to its ability to modulate the HPA axis, oxidative stress, and neuroinflammatory responses. In fact, GSE administration markedly reduced RS levels in the prefrontal cortex, hippocampi, and small intestine of stressed mice, concomitant with a decrease in plasma corticosterone concentrations. Moreover, GSE downregulated the mRNA expression of the proinflammatory cytokines INF-γ, IL-1β, and NF-κB in these same tissues. Overall, these results suggest that the behavioral improvements elicited by GSE are closely associated with its capacity to attenuate oxidative stress and neuroinflammation triggered by immobilization stress.

Our research group, in agreement with previous studies [20, 26, 27], has characterized the ARS paradigm as a reliable model of stress-induced depressive-like pathology, which encompasses both emotional and physical components and disrupts the brain’s intracellular redox balance. Consistent with our findings, animals exposed to ARS exhibited a marked increase in immobility time in the TST, reflecting a depressive-like phenotype. Pretreatment with GSE significantly reduced this immobility time, in line with previous findings by Jiang et al. [19], who reported that GSE at 20 and 40 mg/kg decreased immobility in an LPS-induced depressive-like behavior model. A modest reduction in immobility time was also observed in GSE-treated non-stressed mice, suggesting that GSE may exert intrinsic neuromodulatory effects under basal conditions. Similar findings have been reported previously, supporting the hypothesis that the polyphenolic constituents of GSE may influence behavioral responses independently of stress exposure [19]. Importantly, no alterations in locomotor activity were observed in the OFT in either the stressed or non-stressed groups, confirming that the reduced immobility was not attributable to nonspecific changes in general activity.

The behavioral assessment performed in the present study was designed to evaluate antidepressant-like behavior while controlling for possible locomotor effects. Future studies incorporating complementary behavioral paradigms, such as the sucrose preference test, may further expand the behavioral characterization of GSE and provide additional insight into its antidepressant-like profile.

The brain is highly susceptible to fluctuations in glucocorticoid levels, and even brief episodes of stress can elicit adverse neurobiological responses [28]. Short-term HPA axis hyperactivation may disrupt neuronal homeostasis, particularly in prefrontal and hippocampal circuits involved in emotional and cognitive regulation [29]. In humans, cortisol represents the main glucocorticoid released during stress [30], whereas corticosterone serves as the principal equivalent in rodents, making its quantification a reliable biomarker of HPA axis activation [31]. Here, GSE pretreatment reduced plasma corticosterone levels in stressed mice, suggesting that its antidepressant-like mechanism involves modulation of HPA axis activity.

It is well established that elevated corticosterone levels are closely linked to oxidative damage in the brain [18, 32, 33]. Excessive activation of glucocorticoid receptors disrupts mitochondrial function, leading to an imbalance between RS generation and endogenous antioxidant defenses. This imbalance promotes lipid peroxidation, protein oxidation, and DNA damage, ultimately impairing neuronal integrity and synaptic plasticity [18]. Brain regions with high glucocorticoid receptor density, such as the hippocampi and prefrontal cortex, are particularly vulnerable to these effects, contributing to the neurobiological alterations observed in stress-related mood disorders [28]. Therefore, the hippocampi and prefrontal cortex were specifically investigated because they are key components of the corticolimbic network involved in emotional regulation and stress adaptation and are particularly susceptible to stress-induced oxidative stress and neuroinflammation [34–37]. While the hippocampi primarily encodes contextual information, modulates HPA axis activity, and supports memory formation, the prefrontal cortex exerts top-down executive control over emotional responses and behavioral adaptation [35]. Stress exposure can disrupt communication within this hippocampal–prefrontal circuit through glucocorticoid dysregulation, oxidative stress, impaired synaptic plasticity, and neuroinflammatory processes, contributing to cognitive dysfunction and depressive-like behaviors [36, 37]. Therefore, the simultaneous modulation of oxidative and inflammatory markers in both regions by GSE may reflect a coordinated protective effect on stress-responsive neural pathways rather than isolated actions on individual brain structures.

In the present study, ARS increased corticosterone levels and RS generation in the hippocampi, prefrontal cortex, and small intestine, confirming the close association between glucocorticoid elevation and oxidative stress. Conversely, GSE pretreatment significantly reduced RS levels in all analyzed regions, reinforcing its antioxidant potential. These results align with those of Jiang et al. [19], who observed similar antioxidant and neuroprotective effects of GSE in the hippocampi of prenatally stressed female rats. Importantly, this study extends previous findings by demonstrating for the first time that GSE also attenuates oxidative stress in the prefrontal cortex and small intestine of female mice, highlighting its protective effects on both central and peripheral stress-sensitive tissues.

Oxidative stress is a key modulator of inflammatory responses, establishing a reciprocal relationship with neuroinflammation [38]. Although distinct, these processes are mechanistically interconnected and mutually amplified. During oxidative injury, damaged cellular components act as danger-associated molecular patterns (DAMPs), which activate innate immune receptors and trigger inflammatory signaling in the brain [39]. These cascades stimulate the TLR4/NF-κB pathway, leading to the upregulation of proinflammatory cytokines such as IL-1β [40]. Once activated, NF-κB induces the expression of cytokines and enzymes that generate reactive oxygen and nitrogen species, thereby perpetuating oxidative damage and neurotoxicity [39] This self-sustaining cycle contributes to the overproduction of neurotoxic mediators and cytokines, ultimately promoting neuronal dysfunction and depressive-like behaviors.

In a previous study, Rigotti et al. [7], investigated the anti-inflammatory potential of the same GSE used here through in silico network pharmacology and molecular docking analyses. Chemical characterization by HPLC-DAD identified six phenolic compounds, with catechin and rutin as the predominant constituents. Computational screening revealed strong interactions between these molecules and key targets within the TLR4 signaling pathway, particularly AKT1 and MAPK8, which regulate NF-κB activation. Corroborating these findings, the present study demonstrated that ARS increased NF-κB and IL-1β mRNA expression, whereas GSE pretreatment significantly reduced both markers in the hippocampi, prefrontal cortex, and small intestine, suggesting attenuation of inflammatory signaling pathways in central and peripheral tissues. Considering previous evidence linking GSE to TLR4/NF-κB signaling modulation, this pathway may represent a potential mechanism underlying these effects, although further experimental validation is required.

Additionally, ARS induced significant upregulation of IFN-γ mRNA expression, further supporting the activation of neuroimmune pathways during stress. IFN-γ was selected as an additional inflammatory marker because, beyond its classical role in immune defense, this cytokine has emerged as an important regulator of neuroimmune communication and neuronal function [41]. GSE pretreatment markedly reduced IFN-γ expression in the same regions, indicating a systemic anti-inflammatory effect. These results are consistent with previous evidence that central IFN-γ administration promotes microglial activation, suppresses hippocampal neurogenesis, and induces depressive-like behavior and cognitive deficits [42].

Although IFN-γ contributes to immune surveillance and neuronal homeostasis under physiological conditions, its sustained elevation during stress promotes microglial activation, oxidative stress, impaired synaptic function, and suppression of adult hippocampal neurogenesis, ultimately contributing to cognitive impairment and depressive-like behavior [42, 43]. Moreover, IFN-γ signaling can interact with other inflammatory pathways, including NF-κB activation and IL-1β production, amplifying the neuroinflammatory response and contributing to alterations in stress-responsive brain regions such as the hippocampi and prefrontal cortex. Together, these data suggest that IFN-γ serves as a crucial neuroimmune mediator linking peripheral inflammation to CNS dysfunction in depression and that GSE may exert its antidepressant-like effects, at least in part, by suppressing a coordinated IFN-γ–associated neuroinflammatory response.

Although neurotransmitter imbalances are a central feature of depressive disorders, growing evidence indicates that stress-induced dysregulation of the HPA axis, oxidative stress, and neuroinflammatory signaling represents upstream mechanisms that critically shape monoaminergic and glutamatergic neurotransmission [43–46]. Acute stress exposure triggers glucocorticoid release and inflammatory cascades that precede and modulate neurotransmitter alterations, contributing to the emergence of depressive-like phenotypes [30]. In this context, the present study was designed to investigate these early mechanistic drivers rather than downstream neurotransmitter levels. By demonstrating that GSE attenuates HPA axis hyperactivation, redox imbalance, and neuroinflammatory responses while preventing depressive-like behavior, our findings provide mechanistic insight into how GSE may preserve neurotransmission indirectly. Nevertheless, future studies incorporating direct neurochemical analyses will be important to further elucidate the impact of GSE on specific neurotransmitter systems.

Rigotti et al. [7] also proposed that GSE can possibly modulate the microbiota–gut–brain axis by attenuating intestinal oxidative and inflammatory signaling. Oxidative stress may compromise intestinal barrier integrity, facilitating bacterial lipopolysaccharide translocation and activation of TLR4 receptors, thereby amplifying NF-κB–mediated inflammation. The analyses of intestinal tissues in the present study are particularly relevant, as they provide mechanistic insight into how GSE may protect intestinal integrity and modulate the microbiota–gut–brain axis, a key pathway implicated in depression [47].

Although the gut microbiota was not directly assessed in the present study, previous studies suggest that GSE may potentially exert protective effects on the gut–brain axis through multiple complementary mechanisms. A substantial proportion of dietary polyphenols reaches the colon, where they are metabolized by the gut microbiota into bioactive phenolic metabolites that can locally modulate the intestinal environment [48]. In addition, GSE has been reported to exhibit prebiotic-like effects by promoting the growth of beneficial bacteria, including *Bifidobacterium*, *Lactobacillus*, *Akkermansia*, and *Roseburia*, while reducing opportunistic and pro-inflammatory taxa such as *Streptococcus*, *Proteobacteria*, and *Enterococcus* [48–51].

These microbial changes are accompanied by improved intestinal redox homeostasis, reinforcement of epithelial barrier integrity through preservation of tight junction proteins and mucin expression, and restoration of microbial metabolic balance [48, 51, 52]. Therefore, the attenuation of intestinal oxidative stress and inflammatory markers observed in the present study is compatible with a mechanism in which GSE may contribute to the maintenance of intestinal homeostasis, potentially influencing gut–brain communication and the behavioral and molecular responses observed after ARS exposure.

However, some limitations of the present study should be acknowledged. The absence of microbiota composition analysis limits the understanding of whether changes in intestinal microbial populations directly mediate the observed protective effects of GSE. Likewise, the lack of evaluation of enzymatic antioxidant markers such as superoxide dismutase (SOD), catalase (CAT), and nonenzymatic components such as reduced glutathione (GSH) restricts a more comprehensive interpretation of the oxidative mechanisms underlying GSE action, since it has been associated with the enhanced activity of these enzymes in different research models [53–55].

Additionally, the inclusion of a positive control group in future studies may allow direct comparisons with established interventions and further contextualize the efficacy of GSE relative to known protective agents. Also, the exclusive use of female mice precludes comparisons of sex-dependent responses to GSE, while the lack of estrous-cycle monitoring represents an additional limitation. Future studies including both sexes and systematic estrous-cycle assessment are warranted. Furthermore, although the present study demonstrated molecular and biochemical alterations associated with GSE administration, histopathological analyses were not performed. Future investigations incorporating neuronal integrity assessments may provide additional insights into potential structural changes associated with GSE effects.

Finally, the amygdala was not assessed in the present study; given its central role in emotional regulation and stress-related behaviors, future investigations should include this brain region to provide a more complete neurobiological characterization of GSE’s effects. Future studies integrating microbiota profiling and metabolomic approaches could provide deeper insights into the metabolic and microbial pathways modulated by GSE, elucidating its role in the microbiota–gut–brain axis with greater precision.

Collectively, this study provides in vivo evidence that GSE exerts anti-inflammatory and protective effects against stress-induced alterations, at least in part, through the modulation of inflammatory signaling responses, including reduced NF-κB and IFN-γ expression, contributing to the attenuation of neuroinflammatory responses associated with depressive-like behavior.

## Conclusion

In summary, this study demonstrates that GSE attenuates behavioral, oxidative, and inflammatory alterations induced by acute stress in female mice. The extract was associated with changes in HPA axis activity, redox status, and cytokine signaling in brain and intestinal tissues, which may be consistent with a potential systemic protective effect. These findings suggest that GSE may influence HPA axis activity and inflammatory signaling along the gut–brain axis, warranting further investigation of its potential as a complementary nutritional strategy for stress-related mood disorders.

## Data Availability

All data generated or analyzed in this study are available from the corresponding author upon reasonable request. No publicly accessible repository was used due to the nature of the dataset and institutional restrictions.
